# Association of high-risk comorbidity with overall survival among patients with gastric cancer and its sex-specific differences in China: a retrospective observational cohort study

**DOI:** 10.1186/s12885-023-11374-4

**Published:** 2023-09-28

**Authors:** Ju Wu, Simiao Tian, Jian Xu, Nan Cheng, Xi Chen, Jiajun Yin, Zhequn Nie

**Affiliations:** 1 Department of General Surgery, Affiliated Zhongshan Hospital of Dalian University, Dalian City, 116001 Liaoning Province China; 2 Department of Medical Record and Statistics, Affiliated Zhongshan Hospital of Dalian University, Dalian City, 116001 Liaoning Province China

**Keywords:** Comorbidity, Age-adjusted Charlson comorbidity index, Overall survival, Gastric cancer

## Abstract

**Background:**

Concomitant diseases often occur in cancer patients and are important in decision-making regarding treatments. However, information regarding the prognostic relevance of comorbidities for mortality risk is still limited among Chinese gastric cancer (GC) patients. This study aimed to investigate the association between comorbidities and 3-year mortality risk.

**Methods:**

This retrospective study enrolled 376 GC patients undergoing radical gastrectomy at the Affiliated Zhongshan Hospital of Dalian University from January 2011 to December 2019. Demographic and clinicopathological information and treatment outcomes were collected. Patients were divided into low-, moderate- and high-risk comorbidity groups based on their Charlson Comorbidity Index (CCI) and age-adjusted CCI (ACCI) scores. Kaplan-Meier survival and Cox regression analyses were used to examine 3-year overall survival (OS) and mortality risk for each group.

**Results:**

The median follow-up time was 43.5 months, and 40.2% (151/376) of GC patients had died at the last follow-up. There were significant differences in OS rates between ACCI-based comorbidity groups (76.56; 64.51; 54.55%, log-rank *P* = 0.011) but not between CCI-based comorbidity groups (log-rank *P* = 0.16). The high-risk comorbidity group based on the ACCI remained a significant prognostic factor for 3-year OS in multivariate analysis, with an increased mortality risk (hazard ratio [HR], 1.99; 95% CI, 1.15–3.44). Subgroup analysis revealed that this pattern only held for male GC patients but not for female patients.

**Conclusion:**

The present study suggested that high-risk comorbidities were significantly associated with a higher mortality risk, particularly in Chinese male GC patients. Moreover, the ACCI score was an independent prognostic factor of long-term mortality.

**Supplementary Information:**

The online version contains supplementary material available at 10.1186/s12885-023-11374-4.

## Introduction

Gastric cancer is one of the most commonly diagnosed cancers and the third leading cause of cancer-related deaths worldwide, accounting for more than 1 million new cases and more than 783,000 related deaths each year [1, 2]. During the past two decades, the burden of gastric cancer in China has been remarkably high, with 612,820 new cases and an age-standardized mortality rate of 21.72 per 100,000 in 2019 [3]. Moreover, given the aging of the population, increased life expectancy and lifestyle changes, China is now experiencing a significantly higher incidence of early-onset gastric cancer and a higher mortality/incidence ratio than most developed countries [3]. More importantly, the proportion of elderly patients diagnosed with gastric cancer has recently shown an increasing trend.

The prognosis of gastric cancer is especially poor for elderly patients as they are more likely to have a reduced functional reserve [4, 5] and more comorbidities [6]. A comorbidity refers to the presence of one or more health conditions or disorders concomitant with a primary disease such as cancer [7]. Several studies have shown that comorbidities could affect cancer treatment options, and they are associated with a higher risk of adverse short- and long-term outcomes, including postoperative complications, morbidity and mortality [8, 9]. However, a comprehensive evaluation of the impact of various comorbid diseases is difficult to perform in clinical practice, and optimal tools have not been sufficiently established. Therefore, several tools have been developed to evaluate the degree of comorbidity burden, such as the Charlson comorbidity index (CCI) [10], its extensions [11, 12] and others [13].

In 1984, Charlson et al. proposed and developed the CCI to evaluate the impact of preoperative comorbidities on a variety of cancers and medical disorders [10]. This index is a weighting system that incorporates 19 different medical conditions, with each condition being assigned a weight according to its impact on mortality. Then in 1994, Charlson et al. extended and established a new scoring system, the aged-adjusted CCI (ACCI), which combined age and comorbidities to more accurately predict operative mortality [11]. The CCI and ACCI are widely used in predicting both the short- and long-term outcomes of various malignant tumours [14–16], and investigators have recently shown a better utility of the ACCI than the CCI for mortality and postoperative complications [12, 17, 18].

Although the effect of the CCI-type defined comorbidity on the prognosis of patients with GC has been reported in previous studies [17, 19, 20], evidence on the association of comorbidity with the prognosis of Chinese patients with GC is still scarce. Therefore, in the present study, we aimed to assess the association between comorbidities and short-term mortality risk and investigate whether there are sex-specific differences in the association among Chinese patients with GC.

## Methods

### Study population and data collection

This was a single-centre, retrospective study. Patients diagnosed with primary gastric cancer and who were treated with radical gastrectomy at the Affiliated Zhongshan Hospital of Dalian University between January 2011 and December 2019 were enrolled. Information on clinicopathological data and treatment outcomes was extracted from electronic medical records. Among them, patients’ baseline demographic data, comorbidities, date and type of surgery and tumour characteristics (i.e., size, location, morphology, appearance, histology, and depth of invasion) were collected. This study was approved by the Ethics Committee of Affiliated Zhongshan Hospital of Dalian University, and was conducted in accordance with the Declaration of Helsinki. Due to the nature of the retrospective design, informed consent of the patients was waived and granted by the Ethics Committee of Affiliated Zhongshan Hospital of Dalian University.

### Clinicopathological characteristics

All clinicopathological variables were determined according to the same guidelines and included tumour size, lesion location, tumour-node-metastasis (TNM) staging, histologic type, lymphovascular invasion, vertical margin and adjuvant chemotherapy. The TNM staging of all patients was categorized according to the 8th edition of the American Joint Committee on Cancer (AJCC) Staging Manual criteria [21]. Pathological tumour (pT) and lymph node (pN) stages were evaluated by pathological assessment after surgery. Lesion locations were classified into upper, lower, middle and mixed positions. Histologic type was classified into well, moderately and poorly differentiated adenocarcinoma; lymphovascular invasion was defined as the observable spread of tumour cells through the lymphatic vessels.

### Follow-up for death

All of the patients were actively followed up after surgery by physical examination, laboratory tests and imaging examinations every 3 months for the first 2 years, every 6 months for the next 3 years, and annually thereafter. The end point of this study was overall survival (OS), which was measured from the date of primary surgery to the date of death from any cause or last follow-up (August 14th, 2022).

### Measurement of comorbidities

Information on patients’ comorbidities was obtained by using secondary and other diagnoses and was assessed by using all the available information from patients’ detailed electronic health records in primary care, outpatient, and in-patient hospital information during the period from 3 months before to 3 months after cancer diagnosis. Then, these comorbidities were identified by using an algorithm based on International Classification of Diseases, Tenth Revision (ICD-10) codes, were assessed rigorously by qualified special physicians. The comorbidity measure used in this study included the following medical conditions: myocardial infarction, congestive heart failure, peripheral vascular disease, dementia, cerebrovascular disease, chronic pulmonary disease, ulcer disease, diabetes, hypertension, mild liver disease, moderate or severe renal disease, hemiplegia, malignant lymphoma, any tumor, moderate or severe liver disease, metastatic solid tumor, acquired immune deficiency syndrome. In this study, we used the CCI and ACCI to assess comorbidities. The CCI scores are defined by the summarized score based on 17 medical conditions with their corresponding weight ranging from 1 to 6 points [10] (Supplementary Table 1), whereas ACCI scores, a combination of the age equivalence index and CCI, are calculated with additional points added for age, with assigning each decade of age over 40 years a CCI score of 1 (e.g., 50–59 years, 1 point; 60–69 years, 2 points; and 70–79 years, 3 points) [11].

### Statistical analyses

Continuous data with normal distributions are presented as the mean and SD, whereas those not normally distributed are presented as the median and IQR after assessing normality by the Shapiro-Wilk test. Categorical data are presented as frequencies and percentages. The optimal clinical cut-off values of the CCI and ACCI were determined according to the lowest log-rank P value from the Kaplan-Meier survival analysis by using X-tile software (Version 3.6.1, Yale University School of Medicine) [22]. Thus, patients were categorized into three comorbidity groups (CCI and ACCI category): a low-risk (CCI = 0; ACCI = 0–2), a moderate-risk (CCI = 1; ACCI = 3–4), and a high-risk comorbidity group (CCI≥2; ACCI≥5). The comparison between vital status was performed using a t-test or Kruskal-Wallis test, where appropriate, for continuous variables and a ${\chi }^{2}$ test for categorical variables. Unadjusted survival proportion rate was estimated using the Kaplan-Meier method and compared using the log-rank test. Cox regression analysis was applied to examine the association between OS and comorbidities, with time since surgery as the underlying timescale. The adjusted covariates were selected based on previously published studies and clinically relevant experience. The associations were reported as hazard ratios (HRs) and their 95% CIs and were first unadjusted (unadjusted model) and further adjusted for sex, lymphovascular invasion, tumour size, lesion location and adjuvant chemotherapy (adjusted model). Age was not taken into account in the model because it was used for the ACCI calculation. All analyses were performed with R statistical software (version 4.1.2; R Foundation for Statistical Computing) [23], and a two-tail P-value < 0.05 was considered statistically significant.

## Results

### Baseline characteristics

A total of 376 patients with gastric cancer were included in this study, among whom, 106 (28%) patients were female, and the mean age was 66.22±10.54 years. The baseline and clinicopathological characteristics are presented in Table 1. The average values of CCI and ACCI were 0.8 and 3.78, respectively, and their differences between men and women were not statistically significant. Among the overall sample, the distributions of patients with GC in the CCI- and ACCI- based comorbidity groups were quite different: when using the ACCI criterion, 56.38% of patients were classified into the moderate-risk comorbidity group, whereas 53.46% had low-risk comorbidities when using the CCI criterion. With regard to sex-specific distributions, consistent distribution patterns were found in men and women, and there were no significant differences between them in either the CCI or ACCI groups (P=0.502 and P = 0.579). Furthermore, none of these clinicopathological characteristics or follow-up periods showed any significant differences between two sex groups.

**Table 1:** Baseline characteristics of studies sample

	All	Men	Women	*P*-value
No. of participants	376	270	106	
Age (years), mean (SD)	66.22 (10.54)	65.66 (9.59)	67.63 (12.59)	0.148
Smoker, n (%)	123 (32.71%)	122 (45.19%)	1 (0.94%)	< 0.001
Alcohol drinker, n (%)	105 (27.93%)	102 (37.78%)	3 (2.83%)	< 0.001
ACCI group, n (%)				0.502
Low-risk comorbidity (0–2)	73 (19.41%)	53 (19.63%)	20 (18.87%)	
Moderate-risk comorbidity (3–4)	212 (56.38%)	156 (57.78%)	56 (52.83%)	
High-risk comorbidity (≥5)	91 (24.20%)	61 (22.59%)	30 (28.30%)	
CCI group, n (%)				0.579
Low-risk comorbidity (0)	201 (53.46%)	141 (52.22%)	60 (56.60%)	
Moderate-risk comorbidity (1)	118 (31.38%)	85 (31.48%)	33 (31.13%)	
High-risk comorbidity (≥2)	57 (15.16%)	44 (16.30%)	13 (12.26%)	
Lesion location, n (%)				0.236
Lower	218 (57.98%)	152 (56.30%)	66 (62.26%)	
Middle	123 (32.71%)	88 (32.59%)	35 (33.02%)	
Upper	21 (5.59%)	17 (6.30%)	4 (3.77%)	
Mixed	14 (3.72%)	13 (4.81%)	1 (0.94%)	
Histologic type, n (%)				0.099
Well differentiated	20 (5.32%)	15 (5.56%)	5 (4.72%)	
Moderate differentiated	144 (38.30%)	112 (41.48%)	32 (30.19%)	
Poor differentiated	212 (56.38%)	143 (52.96%)	69 (65.09%)	
pT, n (%)				0.154
1	64 (17.02%)	41 (15.19%)	23 (21.70%)	
2	42 (11.17%)	32 (11.85%)	10 (9.43%)	
3	27 (7.18%)	16 (5.93%)	11 (10.38%)	
4	243 (64.63%)	181 (67.04%)	62 (58.49%)	
pN, n (%)				0.992
0	139 (36.97%)	99 (36.67%)	40 (37.74%)	
1	74 (19.68%)	54 (20.00%)	20 (18.87%)	
2	69 (18.35%)	50 (18.52%)	19 (17.92%)	
3	94 (25.00%)	67 (24.81%)	27 (25.47%)	
cM, n (%)				0.167
0	349 (92.82%)	247 (91.48%)	102 (96.23%)	
1	27 (7.18%)	23 (8.52%)	4 (3.77%)	
TNM, n (%)				0.277
I	90 (23.94%)	61 (22.59%)	29 (27.36%)	
II	65 (17.29%)	49 (18.15%)	16 (15.09%)	
III	198 (52.66%)	140 (51.85%)	58 (54.72%)	
IV	23 (6.12%)	20 (7.41%)	3 (2.83%)	
Vertical margin, n (%)	4 (1.06%)	3 (1.11%)	1 (0.94%)	1
Lymphovascular invasion, n (%)	150 (39.89%)	114 (42.22%)	36 (33.96%)	0.176
Adjuvant chemotherapy, n (%)	170 (45.21%)	130 (48.15%)	40 (37.74%)	0.087
Survival status				0.341
Alive	225 (59.84%)	157 (58.15%)	68 (64.15%)	
Dead	151 (40.16%)	113 (41.85%)	38 (35.85%)	
Tumor size, mean (SD)	4.54 (2.62)	4.53 (2.58)	4.58 (2.72)	0.85
ACCI scores, mean (SD)	3.78 (1.72)	3.76 (1.63)	3.81 (1.94)	0.821
CCI scores, mean (SD)	0.80 (1.35)	0.83 (1.34)	0.74 (1.39)	0.568
Follow up (month), mean (SD)	49.56 (34.81)	49.27 (34.44)	50.29 (35.88)	0.802

**Fig. 1:**
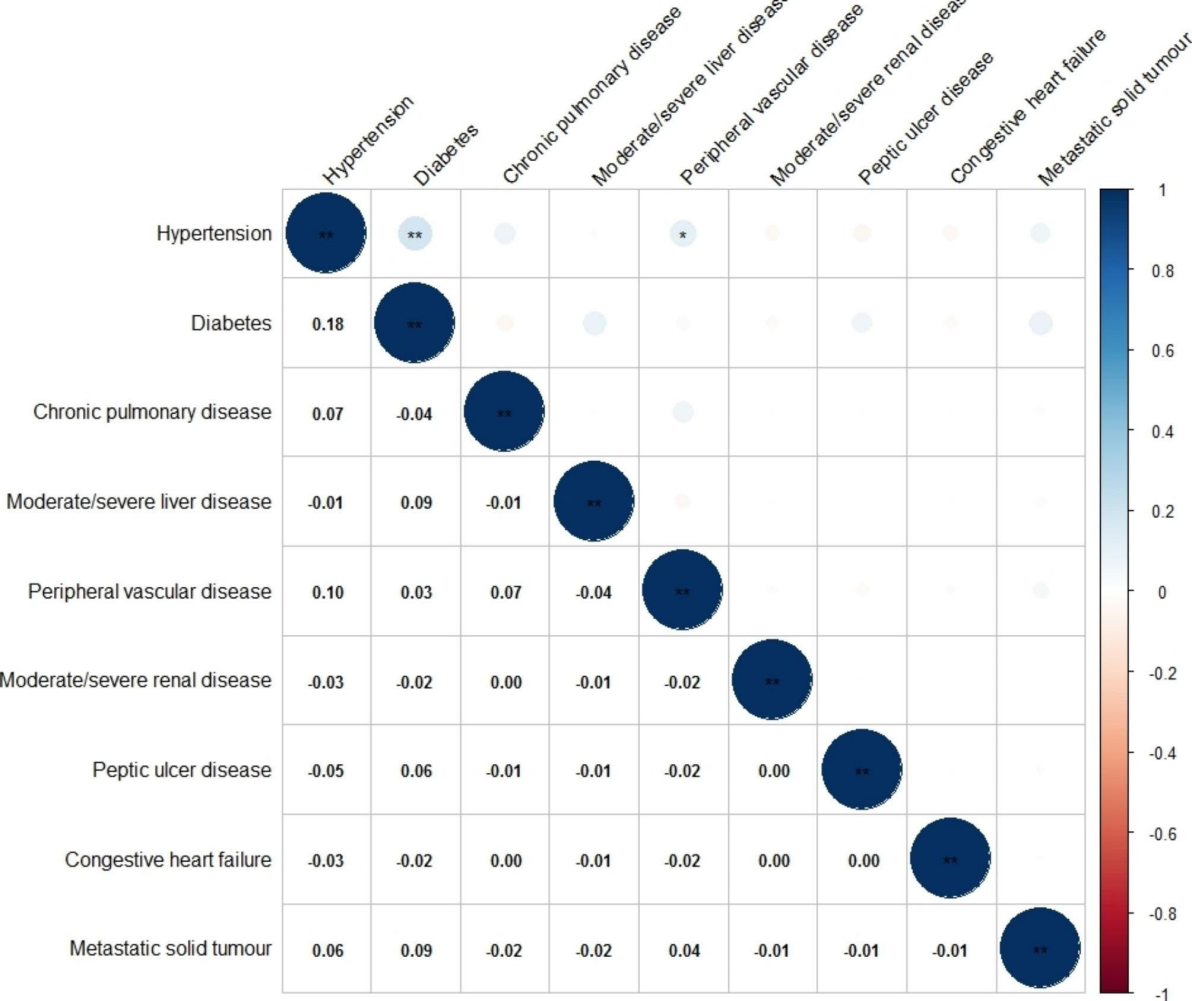
Pearson’s correlogram of comorbidities among Chinese patients with gastric cancer (n = 376). Pearson’s correlation two-side significance value set at *P* < 0.01 and 0.05 for the pairwise correlations between the most common comorbidities, and represented by ** and *, respectively

Among patients with gastric cancer, 111 were diagnosed with hypertension, the most prevalent comorbidity (29.52%), followed by diabetes (17.29%) and heart failure (10.37%) (Table 2). The most frequent pairwise combination was hypertension and diabetes (8.2%), followed by hypertension and heart failure (4.5%). Meanwhile, among those with ≥2 comorbidities, 7 patients had malignant tumours. Figure 1 shows the pattern of pairwise correlations between the most common comorbidities with the two-sided P value set at 0.01 and 0.05 (Fig. 1). The highest pairwise correlation was between hypertension and diabetes (r = 0.18, P <0.001), followed by the correlation between Peripheral vascular disease and hypertension (r = 0.1, P = 0.04).

**Table 2:** Incidence of each comorbidity and distribution of age and additional score CCI score

Comorbidities	n	%	CCI score
Hypertension	111	29.52%	1
Diabetes	65	17.29%	1
Chronic pulmonary disease	3	0.80%	1
Peripheral vascular disease	39	10.37%	1
Peptic ulcer disease	2	0.53%	1
Congestive heart failure	1	0.27%	1
Moderate/severe renal disease	1	0.27%	2
Moderate/severe liver disease	4	1.06%	3
Metastatic solid tumour	11	2.93%	6
Age (year)			
<49 year	7	1.86%	0
50–59 year	15	3.99%	1
60–69 year	84	22.34%	2
70–79 year	144	38.3%	3
≥80 year	126	33.51%	4

### Overall survival

**Fig. 2:**
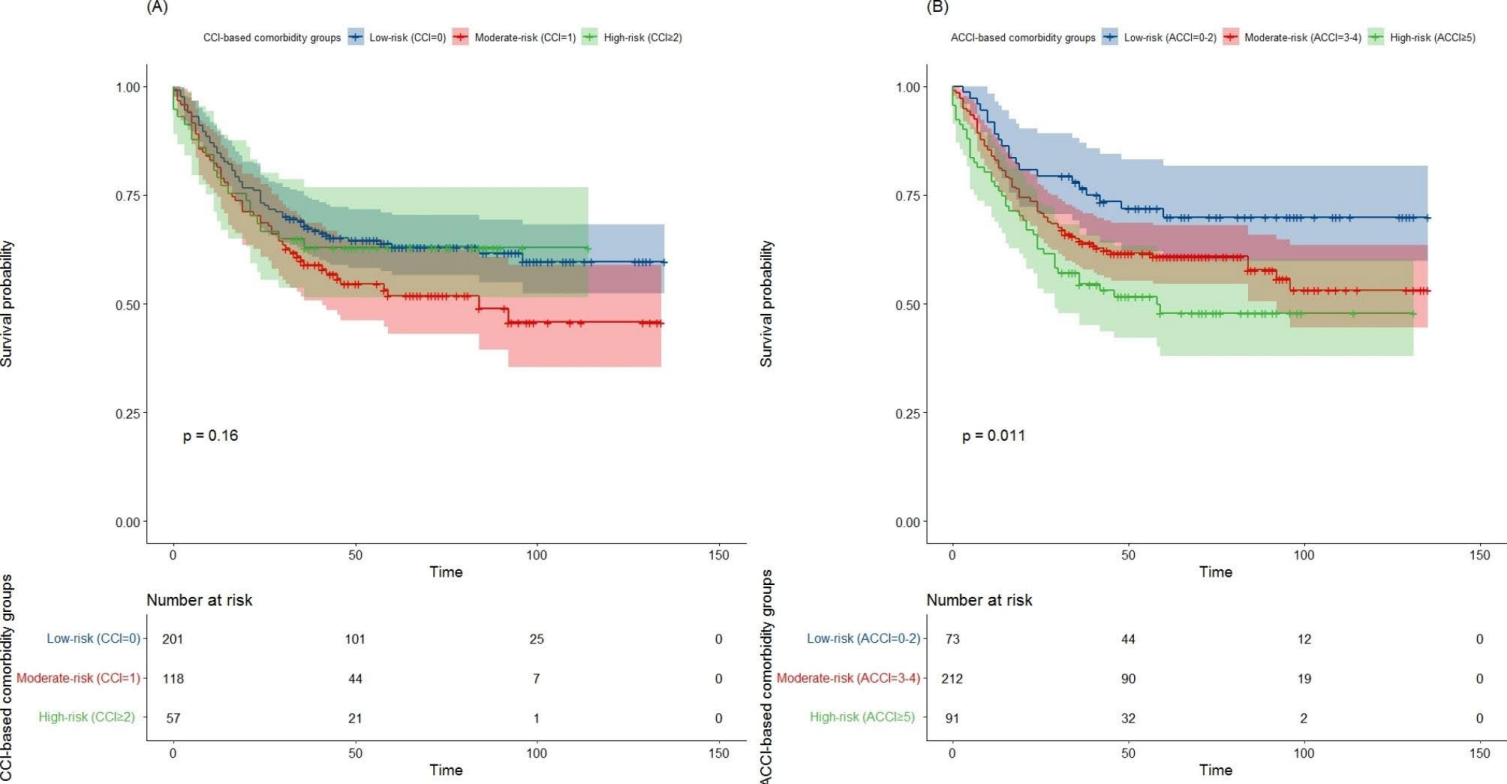
Kaplan-Meier curves comparing the 3-year overall survival of gastric cancer patients with different risk of comorbidities, according to (A) CCI and (B) ACCI staging

The median follow-up time was 43.5 months (range, 0 to 135) and 40.2% (151/376) of patients with gastric cancer had died at the last follow-up. The 3-year OS rates were 76.56% (95% CI 67.4%-86.97%), 64.51% (95% CI 58.36%-71.30%), and 54.55% (95% CI 45.14%-65.91%) for patients in the low-, moderate- and high-ACCI groups, respectively. The 3-year OS of patients with a higher ACCI was significantly lower than that of their counterparts with a lower ACCI (log-rank *P* = 0.011) (Fig. 2A). The respective OS rates for the low-, moderate-, and high-CCI groups were 68.1%, 59.03%, and 62.88%, as shown in Fig. 2B; however, there was no statistically significant difference in OS in the CCI group (log-rank *P* = 0.16).

**Fig. 3:**
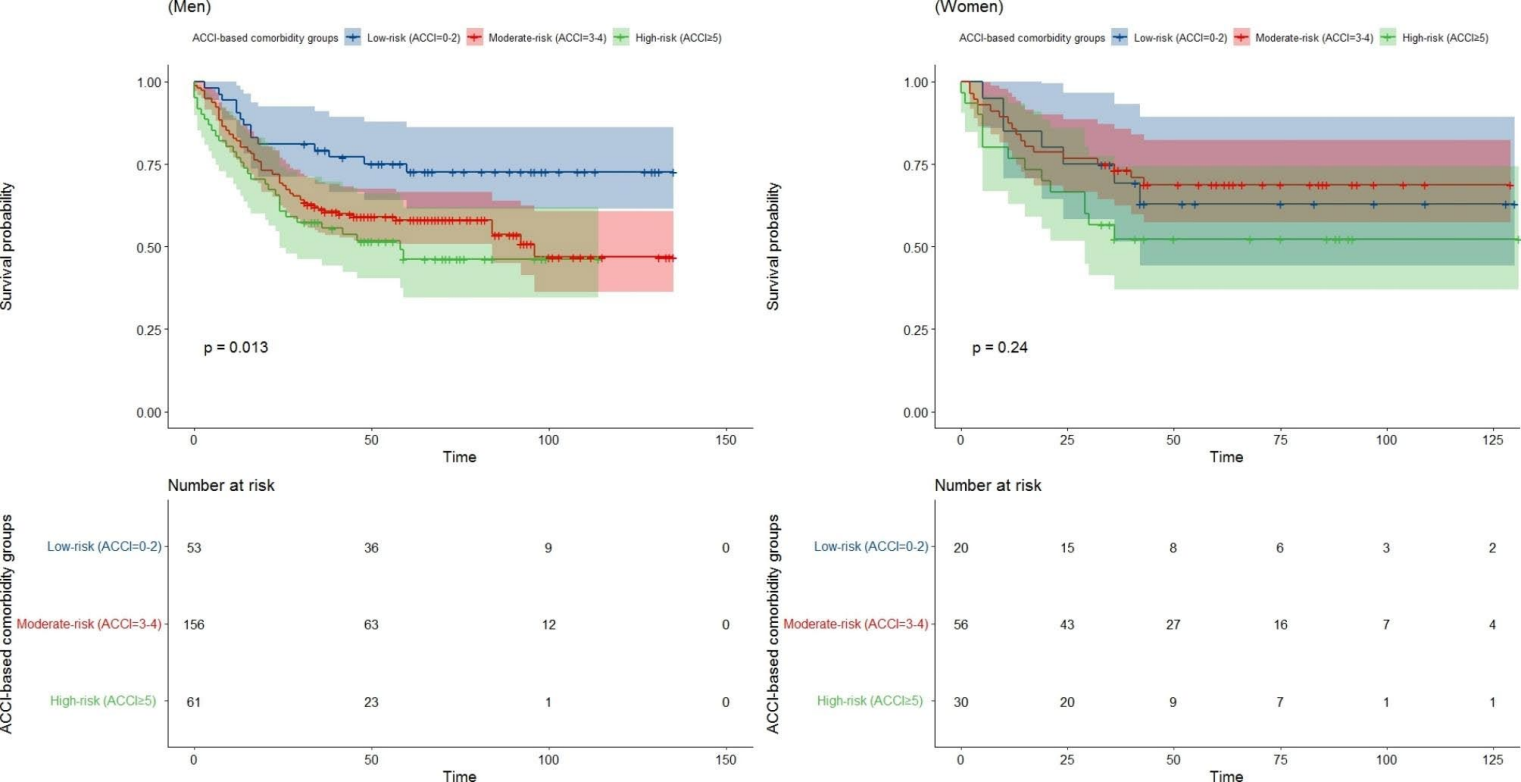
Kaplan-Meier curves comparing the 3-year overall survival of gastric cancer patients with ACCI-based comorbidity groups, stratified by sex. (A) Men and (B) women. Abbreviations: ACCI: age-adjusted Charlson comorbidity index

Subgroup analyses were further conducted by sex. In men, a significant difference was found in OS between the low-, moderate- and high-ACCI groups (3-year OS: 79.2%, 61.4% and 55.53%, respectively; P = 0.013); however, this difference did not reach statistical significance between the three ACCI groups among women (3-year OS: 69.2%%, 73.17% and 52.31%, respectively; P = 0.24) (Fig. 3).

### Association between the ACCI and mortality

The association between comorbidities and mortality was investigated and is presented in Table 3. When using the ACCI, the crude HRs for 3-year OS were 1.6 (95% CI, 0.97–2.5; *P* = 0.064) for patients with moderate-risk comorbidities and 2.2 (95% CI, 1.29–3.6; *P* = 0.004) for patients with high-risk comorbidities when compared with their counterparts with low-risk comorbidities. After adjusting for sex, lymphovascular invasion, tumour size, lesion location and adjuvant chemotherapy, the high-risk comorbidity group was still significantly associated with increased mortality risk for 3-year OS, with an adjusted HR of 1.99 (95% CI, 1.15–3.44). In contrast, both crude and adjusted analyses of overall survival at 3 years found no differences among the three comorbidity groups defined by CCI (crude HR = 1.1, 95% CI, 0.68–1.8 and adjusted HR = 1.00, 95% CI, 0.61–1.65 for the high-risk CCI group, respectively) (Table 3).

**Table 3:** Crude and adjusted hazard ratios of 3-year mortality among Chinese patients with gastric cancer according to CCI and ACCI scoring system

	Crude HR	95%CI	*P*-value	Ajusted HR^a^	95%CI	*P*-value
CCI-based model						
Low-risk group	Reference			Reference		
Moderate-risk group	1.4	0.99-2.0	0.057	1.46	1.02–2.08	0.037
High-risk group	1.1	0.68–1.8	0.684	1.00	0.61–1.65	0.994
ACCI-based model						
Low-risk group	Reference			Reference		
Moderate-risk group	1.6	0.97–2.5	0.064	1.48	0.9–2.43	0.12
High-risk group	2.2	1.29–3.6	0.004	1.99	1.15–3.44	0.014

^a^Adjusted for sex, lesion location, tumour size, lymphovascular invasion and adjuvant chemotherapy

Abbreviations: CCI, Charlson comorbidity index; ACCI, age-adjusted Charlson comorbidity index; HR, hazard ratio; CI, confidence interval

Further subgroup analyses were conducted for ACCI-based comorbidity groups because of the significant associations observed above. When stratifying by sex, the associations varied substantially (Table 4). Moderate- and high-risk comorbidity groups were significantly associated with an increased overall mortality rate compared with the low-risk comorbidity group in men (HR, 1.96; 95% CI, 1.07–3.60, and HR, 2.39, 95% CI, 1.21–4.73, respectively); however, the pattern among women was not significant for either moderate-risk or high-risk comorbidity groups (P = 0.28 and 0.566, respectively).

**Table 4:** Adjusted hazard ratios of 3-year mortality among Chinese patients with gastric cancer stratified by sex

	Men			Women		
ACCI group	aHR	95% CI	*P*-value	aHR	95% CI	*P*-value
Low-risk group	Reference			Reference		
Moderate-risk group	**1.96**	**1.07–3.60**	**0.029**	0.59	0.23–1.53	0.28
High-risk group	**2.39**	**1.21–4.73**	**0.012**	1.32	0.51–3.39	0.566

Model adjusted for lesion location, tumour size, lymphovascular invasion and adjuvant chemotherapy

Abbreviations: ACCI, age-adjusted Charlson comorbidity index; HR, hazard ratio; CI, confidence interval

Abbreviations: CCI: Charlson comorbidity index; ACCI: age-adjusted Charlson comorbidity index

## Discussion

In this hospital-based cohort study, the findings of this study confirm that comorbidities have a significant impact on the OS of Chinese patients with GC, and more importantly, ACCI-measured comorbidities were independent prognostic factors of long-term mortality. Our results highlight the utility of the ACCI as an indicator for OS among patients with GC. To improve the survival of patients with GC who undergo radical gastrectomy treatment, assessing ACCI might be a feasible and useful option for establishing a reasonable treatment strategy for gastric cancer.

Several prospective and retrospective studies have reported the association between the comorbidities/multimorbidities and survival outcomes among patients with GC, but the conclusions have remained inconsistent, some have shown a significant association [20, 24–27], whereas some have not [18, 28]. In a cohort study of 488 Japanese patients with endoscopic submucosal dissection (ESD) for GC after a 5-year follow-up, Tanoue et al. showed that patients with severe comorbidities, defined by the ASA-PS classification, had significantly shorter survival than their counterparts with nonsevere comorbidities (5-year OS rate, 79.1 vs. 87.7%; P < 0.01); furthermore, severe comorbidities were significantly associated with a 2.56-fold increased mortality risk after adjustment for confounders [24]. Consistent findings were confirmed by another three Japanese cohort studies [12, 20, 29], and an inverse association of high-risk comorbidity with the prognosis was observed, with a nearly 8-fold increased risk of mortality, even after adjustment for confounders in both elderly and nonelderly patients [20]. Likely, Cao et al., using a hospital-based cohort of 639 Chinese elderly patients with early gastric adenocarcinoma (EGAC), found an impaired 5-year OS and increased hazard of mortality risk in elderly patients who had CCI ≥ 2 when compared to their counterparts with CCI < 2 (79.7% compared to 94.4%) [25], which was in agreement with results from another Chinese cohort study [30], supporting that the presence of comorbidities is a clinically significant prognostic factor of overall survival. Notably, the authors also showed that age was an important independent risk factor for impaired OS in elderly patients with EGAC, which is consistent with our study in which we used the ACCI to evaluate comorbidities. Similarly, two recent cohort studies that both included Korean elderly early GC patients demonstrated that patients in the high-risk comorbidity group were associated with poorer OS after a long-term follow-up, with a nearly twice higher risk of mortality compared with their counterparts in the low-risk comorbidity group, which suggested its role as independent prognostic factor affecting the survival of elderly GC patients [31, 32]. These consistent and significant results were also reported an a perspective of Western population [26, 33, 34]. For instance, from a recent Swedish cohort including 2154 GC patients, Asplund et al. found that the risk of all-cause mortality was 1.63-fold higher among GC patients with high-risk comorbidity than those with low-risk over a 3-year follow-up after adjusting for demographic, and histopathological confounders [33]. Lombardi et al. analysed a Western multicenter data, which comprised 20 Italian centres and 717 patients with advanced GC, and showed that a positive assocation of high comorbidity burden with greater mortality risk for advanced GC patients who underwent both open and laparoscopic gastrectomy after a median follow-up of 40 months (HR, 1.35; 95% CI, 1.01–1.81) [26]. Similarly, a nationwide study, which comprised 11,196 French patients undergoing esophageal or gastric cancer surgery, also confirmed that patients in the high risk comorbidity group had 3.86-fold increased risk of 30-day postoperative mortality, when compared with their counterparts in the low risk group [27]. Moreover, due to largely limited literature on young GC patients, De et al., using a large cohort of 70,084 American patients, focused specifically on young adults GC patients aged ≤ 40 years and demonstrated that comorbidity was still a significant and strong predictor of 5-year overall survival for this young subgroup of patients [34]. The findings from our hospital-based study added robust evidence to the literature, and confirms a significant increase in 3-year mortality risk among Chinese GC patients with high-risk comorbidities, with a 70% higher risk than their peers with low-risk comorbidities.

While a higher comorbidity burden was found predictive of increased morbidity among patients with GC, irrespective of age groups; however, there are still controversial and conflicting findings with nonsignificant associations observed in Asian [28, 35], Western [36] and Brazilian [37] patients with GC. An early retrospective study including 85 Japanese patients with early GC aged ≥ 85 years revealed no significant difference in 5-year OS rates between patients with ASA-PS-based high and low comorbidities after a median 39-month follow-up, although a significant favourable OS rate was observed for patients without comorbidities when compared with their counterparts with comorbidities [28]. Yang et al. et Shen et al. also showed that despite a higher CCI in elderly Chinese GC patients, comorbidities had not been identified as a prognostic factor for short- and long-term survival [35, 38]. Consistently, a recent Spanish study using data from 591 GC patients found that comorbidity was not associated with postoperative mortality and other short-term inhospital mortalities in the multivariable analysis [36]. Similar nonsignificant results were also observed in two Brazilian cohort studies that high-risk comorbidity defined by CCI was not associated with recurrence and mortality risk [37, 39]. The potential explanation for this discrepancy may be due to the small sample size or short follow-up. Besides, some studies failed to take into account relevant confounders, or dated population-based studies where common comorbidities, such as cardiovascular diseases, were underreported.

Recently, many studies have expanded the ACCI to appraise short- and long-term outcomes in a variety of cancer conditions and reported a promising prognostic role in laryngopharyngeal cancer [19], esophageal cancer [14], prostate cancer [40] and vulvar cancer [15], as well as other severe medical disorders [41, 42]. This study confirms the significant prognostic effect of the ACCI on OS in Chinese patients with GC, in line with previous studies among various populations [12, 18, 30]. In a retrospective study including 122 patients with esophageal cancer after a median follow-up of 6 years, Aoyama et al. found that the OS and RFS rates at 5 years after surgery were 54.4% and 43.6%, respectively, in the low-ACCI group but decreased to 29.2% and 21.3%, respectively, in the high-ACCI group, with adjusted HRs of 1.932 and 2.24, respectively [14]. Koseki et al. and Maezawa et al. demonstrated that the ACCI had a significant prognostic impact on both short- and long-term outcomes among GC patients undergoing curative gastrectomy after a 5-year follow-up [12, 17]. Consistently, to reduce confounding bias, Lin et al. used the propensity score matching method and showed that high-risk comorbidities defined by the ACCI were positively associated with a 1.386-fold increased risk of mortality, indicating that the presence of high-risk comorbidities was an independent risk factor for OS [30].

Moreover, our findings demonstrated that the ACCI score, but not the CCI score, was significantly associated with OS among patients with GC, highlighting the superior clinical influence of the ACCI over the CCI as the comorbidity risk scoring system. Lin et al. reported the ability of the ACCI to predict survival in GC patients after radical gastrectomy, whereas the CCI was not prognostic factor for OS during a median follow-up of 4 years [30]. Similarly, a population-based study, which included 4508 lung cancer patients, also found that the ACCI had better discrimination and predictive accuracy for prognosis in operated lung cancer patients compared with the CCI and ECI, suggesting a merit of widespread applicability for ACCI [18]. Consistently, another retrospective study of 198 patients with laryngopharyngeal cancer revealed a better prognostic effect of ACCI than CCI after a follow-up of 5 years [19]. To some extent, this indicates the importance of age-related physiology for cancer treatment outcomes, and adjusting age to CCI is a reasonable way to evaluate the role of comorbidities.

In the abovementioned studies about the clinical impact of the ACCI on the prognosis in patients with malignant tumours, the way to choose the appropriate ACCI cut-off value was quite discrepant. Some explored the cut-off value on the basis of previous and similar studies that evaluated all potential endpoints [17, 43], while some used a statistical modelling approach [12, 30]. Koseki et al. [12] and Aoyama et al. [14] established the optimal cut-off value according to survival analysis, with the most significant results at ACCI = 4, while Dias-Santos et al. obtained the cut-off point based on the ROC curve method [16]. Inspired by Lin et al.’s study [30] and others, the present study used the X-tile program to determine the ACCI cut-off value from a long-term survival prospective. X-tile software, developed by Robert Camp et al. [22], is a commonly used program that is specific for biomarker cut-off optimization within a time-dependent framework. Thus, we obtained the best cut-off value of the ACCI at 2 and 4 and then divided the whole cohort into a low-risk (ACCI = 0–2), moderate-risk (ACCI = 3–4) and high-risk comorbidity group (ACCI≥5). In addition, a sensitivity analysis was conducted by using the 4-group ACCI classification provided by Yang et al. [18] and similar results were found; we thus combined two moderate groups of ACCI at 3 and 4 into a single moderate group (results not shown). Although there is not yet a consensus on the definitive ACCI cut-off value due to differences among cancer types, patients’ background characteristics, and sample size, a systematic review of the literature suggested that a cut-off value of 3–5 might be useful for the ACCI [14]. Future studies are warranted to clarify the optimum cut-off value for the ACCI.

The present study demonstrated that the ACCI was an independent prognostic factor for OS in patients with GC. One possible explanation is that the ACCI might be associated with postoperative surgical complications. Indeed, recent studies have observed a significant impact of comorbidities on postoperative complications in gastric cancer [17]. For instance, the incidence of surgical complications in the high-ACCI group was more than 1.5 times than in the low-ACCI group among patients with GC [17, 30]. Consistent results have also been observed in other types of malignancies [14, 43, 44]. Kahl et al. showed that, among patients with advanced epithelial ovarian cancer, high ACCI scores were significantly associated with more than a 3.72-fold higher risk of severe postoperative complications than low ACCI scores [43]. Furthermore, Aoyama et al. reported that the incidence of postoperative complications was 27% in the low-ACCI group and 54% in the high-ACCI group [14]. The onset of postoperative complications is associated with decreased survival or an increased risk of disease recurrence in various types of cancers [45–47]. Thus, careful attention should be devoted to the possible development of surgical complications in patients with high ACCI scores when undergoing curative gastrectomy or other surgical treatments for gastric cancer.

The current study further demonstrated sex differences in effect of high-risk comorbidity on overall survival among Chinese GC patients, with a significant association among male patients with GC. To the best of our knowledge, there are few studies with specific focus on this sex-specific effect, and most of previous studies included sex as a relevant confounder. Despite this, consistent results with ours were also reported. Of note, two recent cohort studies found a significant higher mortality risk in Japanese GC men than their female counterparts over a follow-up of 5 years (HR, 1.32, 95% CI 1.04–1.66 and 1.29, 1.06–1.58, respectively) after adjustment for comorbidity and other confounders [12, 17]. A recent study, which used a real-world data from a Western population-based perspective with large sample size, showed the similar results [48]. More precisely, Peltrini et al. investigated changes and perioperative mortality over a 6-year period among 14,512 Italian GC patients who underwent gastrectomies, and they demonstrated that sex was independent risk factors for 90-day mortality, with a 1.14-times increased risk of mortality in men than women [48]. Consistent results of higher mortality risk in male GC patients were also reported in other studies using Asian and European data [6, 24, 26] despite lack of significant differences found. Although the reasons underlying these sex differences are still not justified, but seem complex and multifactorial, a higher proportion of unhealthy lifestyle among Chinese men than women may contribute to this. Indeed, men may have a higher prevalence of gastric cancer risk factors, such as poor hygiene, lower consumption of fruits or vegetables, and higher tobacco consumption and alcohol intake, while women are more attentive to health-related information and behaviors [49]. In the present study, the proportion of smokers and alcohol intake was 45.19% and 37.78% among men, respectively, which were substantially higher than that among women (1 and 3 out 164 female patients). These discrepancies could potentially affect or increase severity of their comorbid conditions, which increases the risk of detecting gastric cancer in late stages and, thus, the mortality risk.

The present study has several limitations that should be mentioned. First, despite being solid in terms of the follow-up period, the present study was retrospective in nature and might suffer from some uncontrolled selection bias. Second, our data were derived from a single institution; further validation with additional datasets is warranted. Third, the present study did not have a large sample size which may lead to a reduced statistical power. In addition, the comorbidity scores were assembled retrospectively and may be underestimated because of missing data. However, in terms of capturing comorbidities, inpatient data and main and contributing diagnoses were included, assuring the sound validity of the ACCI. These abovementioned concerns will be considered in further studies.

## Conclusions

In conclusion, the present study adds evidence to support that the presence of comorbidities, as assessed by the ACCI, was an independent prognostic factor for long-term survival in Chinese GC patients, with an increasing mortality risk associated with a higher ACCI score. To improve the survival rate of GC patients, it is necessary to carefully plan perioperative care and surgical strategies using the ACCI in daily clinical practice.

### Electronic supplementary material

Below is the link to the electronic supplementary material.

Supplementary Material 1

## Data Availability

The data in this study are available from the corresponding author upon reasonable request.
