# Supplementary material for: Association of high-risk comorbidity with overall survival among patients with gastric cancer and its sex-specific differences in China: a retrospective observational cohort study
Source: BMC Cancer. 2023 Sep 28;23:916. doi: 10.1186/s12885-023-11374-4 (PMC10537123; doi:10.1186/s12885-023-11374-4)
Supplement: Supplementary file 1 — Supplementary Material 1 [file 12885_2023_11374_MOESM1_ESM.docx]

Supplementary Table 1. Different weights assigned for specific conditions in the Charlson Comorbidity Index.

| Score | Conditions |
| --- | --- |
| Assigned weights for disease |  |
| 1 | Myocardial infarction |
|  | Congestive heart failure |
|  | Peripheral vascular disease |
|  | Dementia |
|  | Cerebrovascular disease |
|  | Chronic pulmonary disease |
|  | Ulcer disease |
|  | Diabetes |
|  | Hypertension |
|  | Mild liver disease |
| 2 | Moderate or severe renal disease |
|  | Hemiplegia |
|  | Malignant lymphoma |
|  | Any tumor |
| 3 | Moderate or severe liver disease |
| 6 | Metastatic solid tumor |
|  | Acquired immune deficiency syndrome |
